# Supplementary figures and images for: Safety and immunogenicity of intradermal administration of fractional dose CoronaVac®, ChAdOx1 nCoV-19 and BNT162b2 as primary series vaccination
Source: Front Immunol. 2022 Oct 4;13:1010835. doi: 10.3389/fimmu.2022.1010835 (PMC9577032; doi:10.3389/fimmu.2022.1010835)

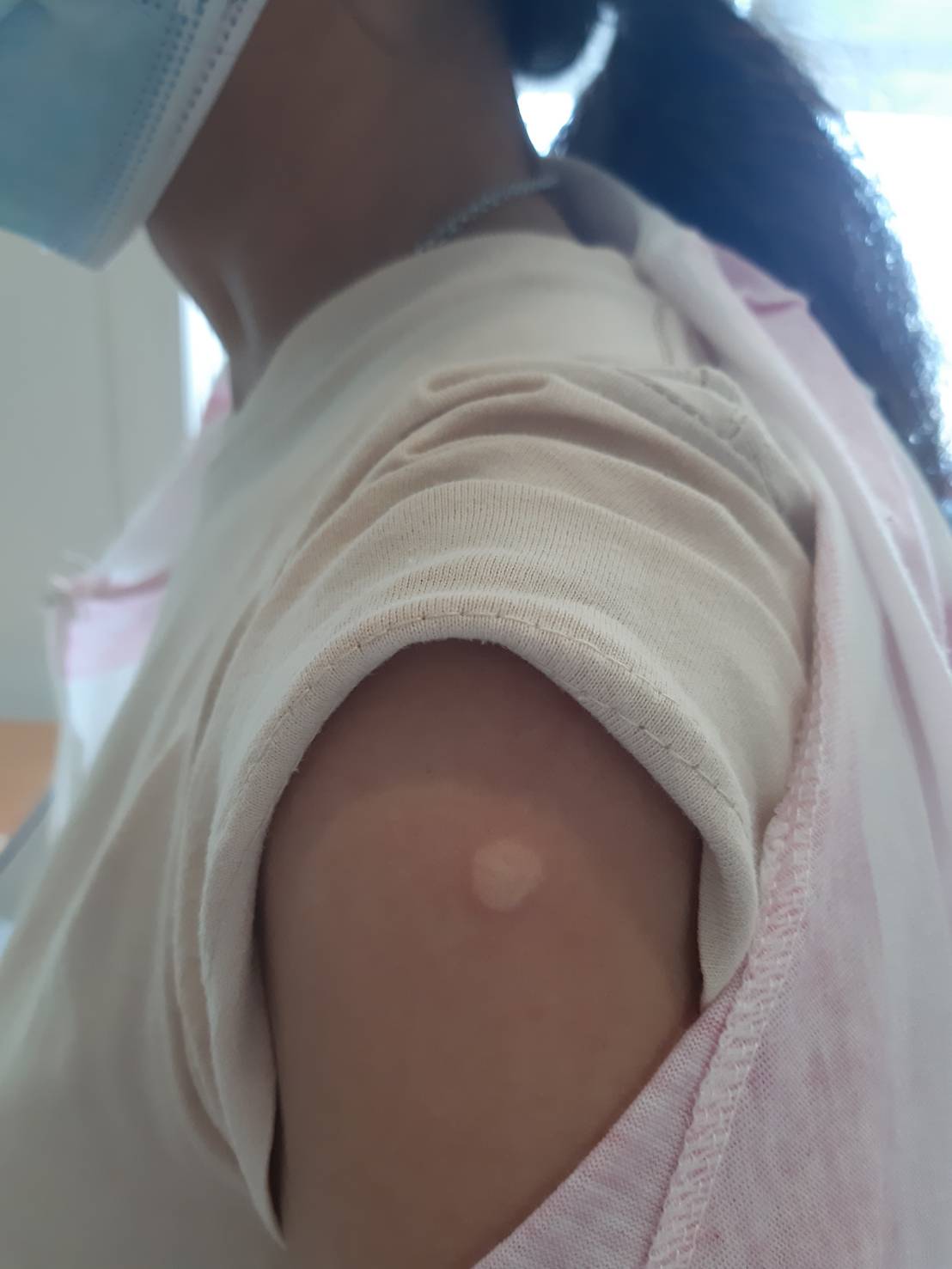

Supplement: Supplementary file 2 [file Image_2.tiff]

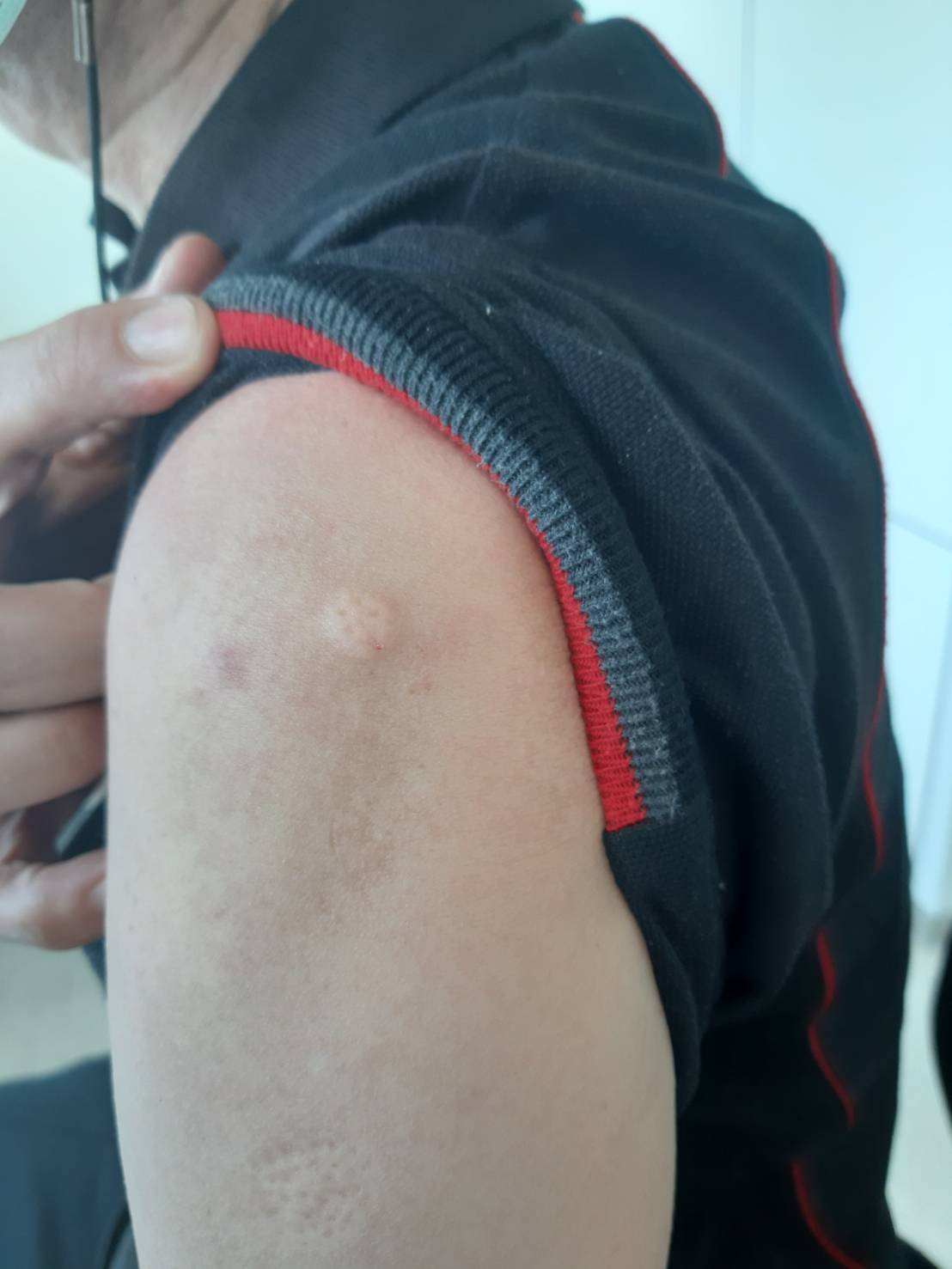

Supplement: Supplementary file 3 [file Image_3.tiff]
