# Supplementary material for: Safety and immunogenicity of intradermal administration of fractional dose CoronaVac®, ChAdOx1 nCoV-19 and BNT162b2 as primary series vaccination
Source: Front Immunol. 2022 Oct 4;13:1010835. doi: 10.3389/fimmu.2022.1010835 (PMC9577032; doi:10.3389/fimmu.2022.1010835)
Supplement: Supplementary file 4 [file Table_1.docx]

**Supplementary Information of A Pilot Study of Safety and Immunological Response against SARS-CoV-2 ancestral and variant strains following Intradermal COVID-19 Administration of**

**CoronaVac®, ChAdOx1 and BNT162b2**

**Supplementary Table 1:** Immunological response following routes and types of COVID-19 vaccines in first and second dose

|  | **Routes and types of vaccines** | | | | | | |
| --- | --- | --- | --- | --- | --- | --- | --- |
| **First dose**  **-**  **Second dose** | **All** | **ID CoronaVac**  **- ID ChAdOx1** | **ID ChAdOx1**  **-**  **ID ChAdOx1** | **ID BNT162b2**  **-**  **ID BNT162b2** | **IM ChAdOx1**  **-**  **ID ChAdOx1** | **IM BNT162b2**  **-**  **ID BNT162b2** | ***P*-value** |
| **Enrolled** | **n=80** | **n=20** | **n=20** | **n=20** | **n=10** | **n=10** |  |
| Excluded from analysis | n=13 | anti-np: positive (n=3)  IgG: positive  (n=1) | anti-np: positive  (n=3)  IgG: positive  (n=2) | anti-np: positive  (n=1)  IgG: positive  (n=2) | anti-np: positive (n=1) |  |  |
| **Anti-RBD IgG (BAU/mL)** | | | | | | | |
|  | **n=67** | **n=16** | **n=15** | **n=17** | **n=9** | **n=10** | ***P*-value** |
| GMC  at baseline (95% CI) | 1.93  (0.13, 0.30) | 0.12  (0.05, 0.28) | 0.27  (0.13, 0.56) | 0.17  (0.07, 0.40) | 0.24  (0.04, 1.50) | 0.26  (0.06, 1.17) | 0.701 |
|  | **n=64** | **n=16** | **n=13** | **n = 17** | **n = 8** | **n =10** | ***P*-value** |
| GMC  at 4 weeks after first dose  (95% CI) | 36.11  (21.35,  61.05) | 5.95  (0.99,  35.94) | 41.76  (23.66,  73.70) | 60.20  (42.19,  85.88) | 74.19  (28.51,  193.05) | 126.13  (79.20,  200.88) | <0.001* |
| GMR between 4 weeks after first dose /baseline  (95% CI) | 204.65  (111.57, 375.38) | 49.70  (8.68,  284.71) | 195.18  (85.61,  444.95) | 356.11  (138.15,  917.92) | 390.41  (67.28,  2265.38) | 487.30  (83.21,  2853.65) | 0.080 |
|  | **n=64** | **n=16** | **n=13** | **n=17** | **n=8** | **n=10** | ***P*-value** |
| GMC  at 2 weeks after second dose (95% CI) | 672.55  (508.74, 889.10) | 748.18  (475.66,  1176.84) | 188.56  (111.06,  320.15) | 1360.89  (1069.84,  17311.11) | 260.90  (119.83,  568.01) | 1906.63  (1441.58, 2521.71) | <0.001* |
| GMR between 2 weeks after second dose /baseline  (95% CI) | 3,811.97  (2323.01, 6255.31) | 6,249.88  (2283.32, 17107.11) | 881.37  (367.22,  2115.40) | 8,050.69  (3389.23, 19123.36) | 1,372.90  (260.16,  7244.83) | 7,366.05  (1572.47, 34505.45) | 0.005* |
| GMR between 2 weeks after second dose /4 weeks after first dose (95% CI) | 18.63  (11.31,  30.69) | 125.75  (27.07,  584.08) | 4.52  (2.71,  7.54) | 22.61  (19.01,  26.88) | 3.52  (1.67,  7.41) | 15.12  (9.79,  23.33) | <0.001* |
|  | **n=49** | **n=14** | **n=6** | **n=17** | **n=3** | **n=9** | ***P*-value** |
| GMC  at 12 weeks after second dose (95% CI) | 161.79  (124.23,  210.72) | 138.21  (93.01,  205.39) | 39.31  (10.99,  140.55) | 230.61  (167.92,  316.71) | 122.17  (87.14,  171.28) | 298.50  (187.96,  474.04) | <0.001* |
| GMR between 12 weeks after second dose /baseline  (95% CI) | 994.26  (574.51, 1720.68) | 1,198.56  (363.83,  3948.45) | 244.72  (41.79,  1433.06) | 1,364.23  (570.85,  3260.25) | 277.74  (2.66,  28960.45) | 1593.11  (345.66,  7342.47) | 0.222 |
| GMR between 12 weeks after second dose /4 weeks after first dose (95% CI) | 3.78  (2.27,  6.29) | 15.43  (3.69,  64.54) | 0.79  (0.29,  2.14) | 3.83  (3.02,  4.86) | 0.53  (0.05,  5.42) | 2.25  (1.26,  4.03) | <0.001* |
| GMR between 12 weeks /2 weeks after second dose  (95% CI) | 0.17  (0.15,  0.19) | 0.17  (0.13,  0.23) | 0.19  (0.11,  0.32) | 0.17  (0.14,  0.21) | 0.18  (0.04,  0.75) | 0.15  (0.11,  0.20) | 0.888 |
| **Live virus focus reduction neutralization tests (FRNT_50_)** | | | | | | | |
|  | **n=64** | **n=16** | **n=13** | **n=17** | **n=8** | **n=10** | ***P*-value** |
| GMT against wuhan strain at 2 weeks after second dose  (95%CI) | 117.44  (81.39,  169.45) | 154.61  (72.67,  328.97) | 23.58  (13.22,  42.06) | 179.68  (105.32,  306.85) | 72.22  (20.68,  252.28) | 436.62  (260.76,  731.10) | <0.001* |
| GMT against delta strain at 2 weeks after second dose  (95%CI) | 72.83  (51.44,  103.12) | 89.85  (43.85,  184.12) | 17.81  (10.77,  29.45) | 125.47  (76.07,  206.98) | 29.62  (9.98,  87.88) | 264.63  (155.92,  449.14) | <0.001* |
| GMT against beta strain at 2 weeks after second dose  (95%CI) | 26.74  (18.54,  38.57) | 29.94  (16.54,  54.20) | 13.77  (10.05,  18.87) | 26.89  (8.52,  84.88) | 14.92  (7.35,  30.31) | 83.50  (44.53,  156.59) | <0.001* |
| GMT against omicron strain at 2 weeks after second dose  (95%CI) | 14.39  (12.18,  16.99) | 17.21  (11.05,  26.83) | 10.00  (10.00,  10.00) | 14.43  (10.48,  19.88) | 10.00  (10.00,  10.00) | 23.05  (12.89,  41.21) | 0.011 |
| Number (%) with FRNT_50_ against omicron strain ≤ 1:20 | 47  (73.44) | 10  (62.50) | 13  (100.00) | 12  (70.59) | 8  (100.00) | 4  (40.00) | 0.003 |
| GMR: wuhan/omicron strain at 2 weeks after second dose  (95%CI) | 8.16  (5.99,  11.13) | 8.98  (4.55,  17.73) | 2.36  (1.32,  4.21) | 12.45  (8.10,  19.13) | 7.22  (2.07,  25.23) | 18.94  (11.62,  30.89) | <0.001* |
| GMR: delta/omicron strain at 2 weeks after second dose (95%CI) | 5.06  (3.83, 6.69) | 5.22  (2.96, 9.20) | 1.78  (1.08, 2.95) | 8.69  (5.67, 13.34) | 2.96  (0.99, 8.79) | 11.48  (8.13, 16.20) | <0.001* |
| GMR: beta/omicron strain at 2 weeks after second dose  (95%CI) | 1.86  (1.37,  2.51) | 1.74  (1.21,  2.49) | 1.38  (1.00,  1.89) | 1.86  (0.65,  5.38) | 1.49  (0.73,  3.03) | 3.62  (2.41,  5.43) | <0.001* |
| **ELISPOT responses (SFU/10^6^ cells)** | | | | | | | |
|  | **n=48** | **n=16** | **n=15** | **n=17** | - | - | ***P*-value** |
| ELISPOT-S  GM at baseline (95% CI) | 3.02  (2.02, 4.51) | 1.87  (0.97, 3.58) | 3.13  (1.32, 7.40) | 4.61  (2.37, 8.99) | - | - | 0.172 |
| ELISPOT-NMO  GM at baseline (95% CI) | 3.22  (2.15, 4.80) | 2.80  (1.39, 5.61) | 2.65  (1.09, 6.43) | 4.35  (2.27, 8.33) | - | - | 0.540 |
|  | **n=64** | **n=16** | **n=13** | **n=17** | **n=8** | **n=10** | ***P*-value** |
| ELISPOT-S  GM at 4 weeks after first dose (95% CI) | 17.18  (12.21,  24.18) | 6.55  (3.54,  12.15) | 36.40  (14.64,  90.53) | 16.77  (10.04,  28.03) | 33.69  (8.09,  140.35) | 18.41  (9.34,  36.29) | <0.001* |
| ELISPOT-NMO  GM at 4 weeks after first dose (95% CI) | 3.62  (2.64,  4.97) | 6.20  (2.69,  14.27) | 4.43  (2.28,  8.62) | 3.16  (1.78,  5.61) | 2.59  (1.08,  6.21) | 1.94  (0.85,  4.43) | 0.016 |
|  | **n=54** | **n=12** | **n=11** | **n=16** | **n=8** | **n=7** | ***P*-value** |
| ELISPOT-S  GM at 2 weeks after second dose (95% CI) | 32.32  (22.92,  45.57) | 15.58  (6.90,  35.19) | 31.79  (17.27,  58.51) | 61.47  (35.00,  107.95) | 20.95  (5.18,  84.66) | 43.80  (17.19,  111.60) | 0.041 |
|  | **n=36** | **n=10** | **n=9** | **n=10** | **n=4** | **n=3** | ***P*-value** |
| ELISPOT-NMO  GM at 2 weeks after second dose (95% CI) | 6.79  (4.90,  9.41) | 8.85  (2.72,  28.78) | 6.26  (2.64,  14.86) | 6.35  (2.63,  15.32) | 4.00  (4.00,  4.00) | 9.80  (0.75,  128.78) | 0.659 |

**Note: -** *p ≤ 0.05

- One-way ANOVA with parametric assumptions satisfied was determined *P*-value among those who received BNT162b2, ChAdOx1, and CoronaVac.

- Abbreviation: BAU/mL: binding antibody unit/mL, GMC: geometric mean concentration, GMT: geometric mean titer, GM: geometric mean, GMR: geometric mean ratio, SFU/10^6^ cells: spot forming unit per million cells
