# Supplementary material for: Safety and immunogenicity of intradermal administration of fractional dose CoronaVac®, ChAdOx1 nCoV-19 and BNT162b2 as primary series vaccination
Source: Front Immunol. 2022 Oct 4;13:1010835. doi: 10.3389/fimmu.2022.1010835 (PMC9577032; doi:10.3389/fimmu.2022.1010835)
Supplement: Supplementary file 5 [file Table_2.docx]

**Supplementary Information of A Pilot Study of Safety and Immunological Response against SARS-CoV-2 ancestral and variant strains following Intradermal COVID-19 Administration of**

**CoronaVac®, ChAdOx1 and BNT162b2**

**Supplementary Table 2:** Adverse events

| **Adverse events** | **Routes and types of vaccines** | | | | | | |
| --- | --- | --- | --- | --- | --- | --- | --- |
| **First dose** | **All** | **ID CoronaVac** | **ID ChAdOx1** | **ID BNT162b2** | **IM ChAdOx1** | **IM BNT162b2** | ***P*-value** |
| **Number of subjects** | **n=80** | **n=20** | **n=20** | **n=20** | **n=10** | **n=10** |  |
| **After first dose** | | | | | | | |
| **Local reaction, n (%)**  Mild, n (%)  Moderate, n (%) | 61 (76.25)  55 (68.75)  6 (7.50) | 12 (60.00)  12 (60.00)  0 (0.00) | 17 (85.00)  15 (75.00)  2 (10.00) | 17 (85.00)  15 (75.00)  2 (10.00) | 7 (70.00)  7 (70.00)  0 (0.00) | 8 (80.00)  6 (60.00)  2 (20.00) | <0.001* |
| **Systemic reaction, n (%)**  Mild, n (%)  Moderate, n (%) | 50 (62.50)  42 (84.00)  8 (16.00) | 13 (65.00)  11 (55.00)  2 (10.00) | 12 (60.00)  10 (50.00)  2 (10.00) | 9 (45.00)  7 (35.00)  2 (10.00) | 8 (80.00)  8 (80.00)  0 (0.00) | 8 (80.00)  6 (60.00)  2 (20.00) | <0.001* |
| **Myalgia, n (%)**  Mild, n (%)  Moderate, n (%) | 33 (41.25)  24 (30.00)  9 (11.25) | 3 (15.00)  2 (10.00)  1 (5.00) | 9 (45.00)  7 (35.00)  2 (10.00) | 7 (35.00)  4 (20.00)  3 (15.00) | 6 (60.00)  5 (50.00)  1 (10.00) | 8 (80.00)  6 (60.00)  2 (20.00) | 0.103 |
| **Fatigue, n (%)**  Mild, n (%)  Moderate, n (%) | 32 (40.00)  31 (38.75)  1 (1.25) | 8 (40.00)  8 (40.00)  0 (0.00) | 8 (40.00)  8 (40.00)  0 (0.00) | 7 (35.00)  7 (35.00)  0 (0.00) | 6 (60.00)  5 (50.00)  1 (10.00) | 3 (30.00)  3 (30.00)  0 (0.00) | 0.619 |
| **Headache, n (%)**  Mild, n (%)  Moderate, n (%) | 39 (48.75)  39 (48.75)  0 (0.00) | 9 (45.00)  9 (45.00)  0 (0.00) | 10 (50.00)  10 (50.00)  0 (0.00) | 7 (35.00)  7 (35.00)  0 (0.00) | 7 (70.00)  7 (70.00)  0 (0.00) | 6 (60.00)  6 (60.00)  0 (0.00) | 0.763 |
| **Nausea, n (%)**  Mild, n (%)  Moderate, n (%) | 9 (11.25)  9 (11.25)  0 (0.00) | 0 (0.00)  0 (0.00)  0 (0.00) | 2 (10.00)  2 (10.00)  0 (0.00) | 2 (10.00)  2 (10.00)  0 (0.00) | 4 (40.00)  4 (40.00)  0 (0.00) | 1 (10.00)  1 (10.00)  0 (0.00) | 0.091 |
| **Diarrhea, n (%)**  Mild, n (%)  Moderate, n (%) | 6 (7.50)  6 (7.50)  0 (0.00) | 1 (5.00)  1 (5.00)  0 (0.00) | 2 (10.00)  2 (10.00)  0 (0.00) | 1 (5.00)  1 (5.00)  0 (0.00) | 1 (10.00)  1 (10.00)  0 (0.00) | 1 (10.00)  1 (10.00)  0 (0.00) | 0.983 |
| **Fever, n (%)**  Mild, n (%)  Moderate, n (%) | 7 (8.75)  2 (2.50)  5 (6.25) | 0 (0.00)  0 (0.00)  0 (0.00) | 3 (15.00)  1 (5.00)  2 (10.00) | 1 (5.00)  1 (5.00)  0 (0.00) | 3 (30.00)  0 (0.00)  3 (30.00) | 0 (0.00)  0 (0.00)  0 (0.00) | 0.134 |
| **Second dose** | **Total** | **ID ChAdOx1** | **ID BNT162b2** |  | | | **P-value** |
| **Number of subjects** | **n=75** | **n=46** | **n=29** |  |  |  |  |
| **After second dose** | | | |  | | |  |
| **Local reaction, n (%)**  Mild, n (%)  Moderate, n (%) | 64 (85.33)  64 (85.33)  0 (0.00) | 39 (84.78)  39 (84.78)  0 (0.00) | 25 (86.21)  25 (86.21)  0 (0.00) |  | | | 0.055 |
| **Systemic reaction, n (%)**  Mild, n (%)  Moderate, n (%) | 45 (60.00)  45 (60.00)  0 (0.00) | 26 (56.52)  26 (56.52)  0 (0.00) | 19 (65.62)  19 (65.62)  0 (0.00) |  |  |  | <0.001* |
| **Myalgia, n (%)**  Mild, n (%)  Moderate, n (%) | 24 (32.00)  19 (25.33)  5 (6.67) | 14 (30.43)  12 (26.09)  2 (4.35) | 10 (34.48)  7 (24.14)  3 (10.34) |  |  |  | <0.001* |
| **Fatigue, n (%)**  Mild, n (%)  Moderate, n (%) | 32 (42.67)  31 (41.33)  1 (1.33) | 21 (45.65)  20 (43.48)  1 (2.17) | 11 (37.93)  11 (37.93)  0 (0.00) |  |  |  | <0.001* |
| **Headache, n (%)**  Mild, n (%)  Moderate, n (%) | 27 (36.00)  18 (24.00)  9 (12.00) | 16 (34.78)  10 (21.74)  6 (13.04) | 11 (37.93)  8 (27.59)  3 (10.34) |  |  |  | <0.001* |
| **Nausea, n (%)**  Mild, n (%)  Moderate, n (%) | 6 (8.00)  6 (8.00)  0 (0.00) | 5 (10.87)  5 (10.87)  0 (0.00) | 1 (3.45)  1 (3.45)  0 (0.00) |  |  |  | 0.716 |
| **Diarrhea, n (%)**  Mild, n (%)  Moderate, n (%) | 5 (6.67)  5 (6.67)  0 (0.00) | 2 (4.35)  2 (4.35)  0 (0.00) | 3 (10.34)  3 (10.34)  0 (0.00) |  |  |  | 0.926 |
| **Fever, n (%)**  Mild, n (%)  Moderate, n (%) | 3 (4.00)  1 (1.33)  2 (2.67) | 1 (2.17)  0 (0.00)  1 (2.17) | 2 (6.90)  1 (3.45)  1 (3.45) |  |  |  | <0.001* |

**Note: -** *p ≤ 0.05

- Chi-square test or Fisher’s exact test was determined *P*-value among those who received BNT162b2, ChAdOx1, and CoronaVac.
